# Synthesis and Characterization of Modified Chitosan Materials with Assessment of Their Antibacterial and Antiviral Activities

**DOI:** 10.3390/jfb17040193

**Published:** 2026-04-17

**Authors:** Dhouha Bouzir, Adel Elamri, Khmais Zdiri, Mohamed Hamdaoui, Christelle Delaite, Abdelaziz Lallam, Omar Anis Harzallah

**Affiliations:** 1 Textile Materials and Processes Laboratory (MPTex), National Engineering School of Monastir (ENIM), University of Monastir, Monastir 5019, Tunisia; bouzir.dhouha@gmail.com (D.B.);; 2 Textile Science and TechnologyResearch Center in Monastir (CRSTTM), University of Monastir, Monastir 5019, Tunisia; khmaiszdiri@gmail.com; 3 LPIM (EA 4567), ENSCMu, University of Haute Alsace (UHA), 68093 Mulhouse, France; christelle.delaite@uha.fr; 4 LPMT (UR 4365), ENSISA, University of Haute-Alsace (UHA), 68093 Mulhouse, France

**Keywords:** modified chitosan, quaternary ammonium chitosan, silver nanoparticles, antibacterial, antiviral activity, SARS-CoV-2, coatings

## Abstract

Modified chitosan (Cs) derivatives were synthesized and evaluated as potential antibacterial and antiviral coatings of medical protective equipment (facial masks, gloves, …). Quaternized chitosan (HTCC) and chitosan–silver nanocomposites (Ag/Cs) were successfully prepared, with structural characterization confirming efficient quaternization and uniform incorporation of silver nanoparticles. Antibacterial testing revealed that HTCC exhibited concentration-dependent activity, while Ag/Cs showed strong broad-spectrum antibacterial effects and enhanced thermal stability. Antiviral assays against SARS-CoV-2 demonstrated significant viral inhibition for HTCC6 and Ag/Cs at non-cytotoxic concentrations (6 mg/mL), highlighting the role of cationic charge and nanoparticle inclusion in antiviral efficacy. These findings indicate that the developed chitosan derivatives are promising candidates for sustainable functional coatings on medical devices, offering potential applications in infection prevention.

## 1. Introduction

The growing demand for antibacterial and antiviral functional materials has been significantly reinforced in recent years, particularly following the global outbreak of Coronavirus disease (COVID-19) [1]. This health crisis highlighted the role of protective equipment such as garments, medical nonwovens, and face masks—as potential vectors for microbial persistence and transmission [2]. In healthcare and community settings, conventional medical protective materials generally lack intrinsic antimicrobial activity and may even facilitate microbial adhesion and proliferation under favorable environmental conditions, including moisture and elevated temperature. Consequently, the development of coating materials capable of actively inhibiting bacterial and viral contamination has become a critical challenge for infection control and public health protection [3].

To date, many academic studies on antimicrobial functionalized devices focus on inorganic agents, especially metallic nanoparticles such as silver, copper, or zinc-based compounds, due to their broad-spectrum antimicrobial effectiveness. However, in commercial production, antimicrobial coatings are predominantly treated with organic antimicrobial agents. Despite the high efficacy of metallic nanoparticles, their use raises concerns related to metal ion release, cytotoxicity, environmental accumulation, and regulatory constraints, particularly for prolonged skin contact or disposable medical products [4,5]. These limitations have encouraged increasing interest in bio-based and biocompatible alternatives that combine antimicrobial performance with sustainability and safety.

Among naturally derived polymers, chitosan has attracted considerable attention as a promising candidate for antimicrobial and antiviral functionalization. Chitosan is a cationic polysaccharide obtained from the partial deacetylation of chitin and is well known for its biodegradability, low toxicity, and intrinsic antibacterial and antiviral properties [6]. Under acidic conditions, the amino groups of chitosan are protonated, giving it cationic polyelectrolyte behavior. Its antimicrobial activity is commonly attributed to the presence of protonated amino groups, which interact electrostatically with negatively charged microbial cell membranes, leading to membrane destabilization and cellular leakage. In addition to its antibacterial effects, chitosan has demonstrated antiviral effects against a range of viruses, including enveloped viruses, through mechanisms involving inhibition of viral adsorption, disruption of viral envelopes, or interference with intracellular replication pathways [7].

However, the direct use of native chitosan remains limited by several intrinsic drawbacks. These include poor solubility at neutral pH, variable antimicrobial efficiency depending on molar mass and degree of deacetylation, and insufficient durability under practical use conditions [8,9]. To address these challenges, numerous chemical modification strategies have been proposed to enhance chitosan solubility, cationic charge density, and biological activity. Among them, quaternization of chitosan has proven particularly effective, as it introduces permanent positive charges that improve water solubility over a wide pH range and strengthen electrostatic interactions with microbial and viral structures [10]. In parallel, the incorporation of metallic nanoparticles, especially silver nanoparticles, into chitosan matrices has been widely investigated to exploit synergistic effects between the biopolymer and inorganic antimicrobial agents [11].

Quaternary ammonium chitosan derivatives, such as N-(2-hydroxypropyl)-3-trimethylammonium chitosan chloride (HTCC), have demonstrated enhanced antibacterial and antiviral performances compared to unmodified chitosan. Their antiviral activity has been linked to increased cationic charge density, which promotes strong interactions with viral envelopes and surface proteins [12]. Similarly, chitosan–silver nanocomposites combine the intrinsic antimicrobial properties of chitosan with the well-established broad-spectrum activity of silver nanoparticles, often resulting in improved thermal stability and sustained antimicrobial efficacy [13]. Nevertheless, the relative contribution of chemical substitution degree, nanoparticle incorporation, and polymer physicochemical properties to antibacterial and antiviral performance remains an open question, particularly in the context of medical applications.

In this context, the present work aims to synthesize and systematically characterize modified chitosan materials specifically designed for antibacterial and antiviral coatings. Low-molar-mass chitosan was first subjected to alkaline deacetylation to achieve a high degree of deacetylation, thereby increasing the availability of reactive amino groups. This parameter is fundamental, as the degree of deacetylation directly affects the biological properties of chitosan [14,15], with higher DDA promoting enhanced biological activities. Two complementary modification routes were then explored: (i) quaternization using glycidyltrimethylammonium chloride to obtain HTCC derivatives with different substitution degrees, and (ii) the synthesis of chitosan–silver nanocomposites through in situ formation of silver nanoparticles. The resulting materials were thoroughly characterized in terms of chemical structure, morphology, thermal behavior, and physicochemical properties. Their antibacterial activity against representative Gram-positive and Gram-negative bacteria was evaluated, and their antiviral efficacy was assessed against SARS-CoV-2, alongside cytotoxicity studies to determine their suitability for biomedical applications.

## 2. Materials and Methods

### 2.1. Materials

The low-molar-mass chitosan Cs (Mw = 25 KDa and deacetylation degree DDA = 77%) used in this study was purchased from Sigma-Aldrich (Dorset, UK). The as-received chitosan was deacetylated by an alkaline treatment with caustic soda.

Glycidyltrimethylammonium chloride (GTMAC), silver nitrate (AgNO_3_), reagent grades of acetic acid (AA), acetone, and all other solvents were also obtained from Sigma-Aldrich.

### 2.2. Methods

#### 2.2.1. Chitosan Desacetylation

To increase the DDA, a 15 M NaOH sodium hydroxide solution was prepared. Then, a mass m of 10 g of chitosan was put into 120 mL of the 15 M NaOH solution. Then, it was left stirring for at least 5 h at a temperature of 110 °C. Subsequently, several washes with distilled water were carried out to remove the NaOH until the pH became neutral (pH = 7). At the end, the chitosan solution was filtered using a fritted glass through the vacuum tap. Then, the deacetylated chitosan CsDDA was rinsed again with pure acetone and air dried. This process yielded CsDDA with a yield of approximately 90%.

#### 2.2.2. Synthesis of HTCC

First, 2.5 g of deacetylated chitosan, equivalent to approximately 15.5 mmol of repeating units, was dissolved in 100 mL of aqueous acetic acid solution (100 mL distilled water + 10 mL acetic acid) and stirred for 30 min until completely dissolved. Second, different volumes of GTMAC (5–6–7–8 mL), corresponding to (38.9–46.7–54.5–62.3 mmol), respectively, were added to obtain different degrees of DS substitution ranging from 50% to 80%. These volumes correspond to molar ratios of GTMAC to chitosan repeating units of approximately 2.5, 3.0, 3.5, and 4.0. Then, the resulting mixture was heated and incubated at 55 °C for 18 h with continuous stirring under a reflux condenser so that the solution does not evaporate. The suspension was then centrifuged at 4000 rpm for 10 min to remove unreacted chitosan from the suspension. The product separated from the supernatant was then precipitated in a 1:1 (*v*/*v*) acetone/ethanol solution and centrifuged again at 4000 rpm for 20 min. An ethanol wash was then performed. The resulting precipitate was finally frozen for freeze-drying in a freeze-dryer to obtain a well-dried modified polymer. HTCC (5, 6, 7 and 8) is denoted for the volumes of GTMAC (5, 6, 7 and 8 mL) respectively. HTCC was obtained with a yield higher than 85% and the polymers were characterized by FTIR, ^1^H NMR scanning electron microscopy (SEM), and conductometric titration to confirm successful quaternization and the degree of substitution.

#### 2.2.3. Synthesis of Silver/Chitosan Nanocomposite (Ag/Cs)

To synthesize Ag/Cs nanocomposite, we first solubilized 0.2 g of deacetylated chitosan in 10 mL of 1% acetic acid solution and left to stir without temperature at 250 rpm until the polymer was completely dissolved. Then, 10 mL of 2% AgNO_3_ solution was added dropwise using a syringe and the solution was left to stir for 24 h at 200 rpm. Subsequently, the solution was added dropwise into 25 mL of 50% NaOH using a syringe. After precipitation of Cs and Ag_2_O in the NaOH solution and the formation of brown spheres, the solution was centrifuged for 10 min at 4500 rpm. Finally, the nanocomposites are washed several times with ultra-pure water and dried under vacuum until they have a fixed mass. According to the data reported by Mirda et al. [16], the corresponding values were adopted in the present work. Ag/Cs was obtained with a yield of 88%.

#### 2.2.4. Morphological Analysis by Scanning Electron Microscopy SEM

Morphological analysis of Ag/Cs nanocomposite and HTCC polymers was examined using a JSM-IT100 InTouchScope™ scanning electron microscope (JEOL Ltd., Tokyo, Japan). Samples were prepared by gold plating.

#### 2.2.5. Fourier Transform Infrared Spectroscopy

Fourier Transform Infrared (FTIR) spectroscopy analysis of raw chitosan, deacetylated chitosan (CSDDA) and HTCC biopolymers were recorded using a PerkinElmer Spectrum IR spectrophotometer (PerkinElmer, Waltham, MA, USA). Measurements were performed using the ATR (attenuated total reflectance) technique.

#### 2.2.6. Nuclear Magnetic Resonance

Nuclear Magnetic Resonance (NMR) analysis was carried out using an Ascend™ 500 MHz spectrometer (Bruker, Billerica, MA, USA). Each tested biopolymer sample was dissolved in D_2_O, at a concentration of 30 mg/mL. Only for the NMR tests of unmodified CS, we added a few drops of trifluoroacetic acid (TFA) to improve spectra resolution. All ^1^H NMR tests were performed at 30 °C and 500 MHz.

#### 2.2.7. Quaternization Degree

The measurement of the quaternization degree QD (also called substitution degree SD) informs us about the progress of the HTCC synthesis reaction. Given the overlap of the NMR signals between the protons of the substituent group and the polymer backbone, we were not able to determine the QD from the ^1^H NMR spectra, as previously described in reference [17]. Therefore, QD of HTCC biopolymers were estimated by the conductometric titration method using a standard silver nitrate (AgNO_3_) solution [18,19]. This method consists of titrating the chloride ions (Cl^−^) present in a HTCC solution. The conductivity measurements were carried out using a SevenCompact Duo conductivity meter (Mettler Toledo, Zaventem, Belgium). Initially, a silver nitrate AgNO_3_ solution with a concentration equal to 0.017 M was prepared, and for each HTCC biopolymer a solution with a concentration of 0.1 g/dL was prepared. The conductivity meter probe is immersed in the HTCC solution, then the AgNO_3_ is slowly added and the solution is stirred. The conductivity is noted after adding a precise volume of AgNO_3_. The addition is continued until the conductivity increases and reaches a stable value. The equilibrium point corresponds to the abrupt change in the slope of the conductivity/AgNO_3_ volume plot. Finally, the QD was calculated using the following Alipour’s [20] relation:

(1) $$QD=\frac{1.7\times 10^{-5}\times V_{\mathrm{AgNO}3}}{[\frac{m-1.7\times 10^{-5}\times V_{\mathrm{AgNO}3}\times 151}{\left(161\times DDA)+(203\times DA\right)}]\times DDA}\times 100$$

With:

- V_AgNO3_: Volume of AgNO_3_ at equivalence.
- m: Mass of HTCC present in the solution.
- DDA: Degree of deacetylation of chitosan.
- DA: Degree of acetylation of chitosan.

#### 2.2.8. Thermogravimetric Analysis TGA

Thermogravimetric measurements were carried out using a Q500 thermogravimetric analyzer (TA Instruments, New Castle, DE, USA), capable of operating at temperatures up to 1000 °C. For each experiment, a small amount of sample (approximately 5–20 mg) was accurately weighed and transferred into a platinum pan before being placed in the furnace of the instrument. The thermal program was set to increase the temperature from 25 °C to 800 °C at a constant heating rate of 10 °C min^−1^, using the instrument control software. All measurements were conducted under a continuous nitrogen atmosphere (40–60 mL min^−1^) to maintain inert conditions and avoid oxidative effects. Changes in sample mass were recorded as a function of temperature, allowing the identification of thermal events such as moisture loss, decomposition steps, and structural transformations. This analysis is performed for all HTCC biopolymers and the Ag/Cs nanocomposite.

#### 2.2.9. Antibacterial Assays

The objective of this study is to evaluate the antimicrobial activity of deacetylated chitosan CsDDA and modified chitosan materials (HTCCs and Ag/Cs) using two experimental methods. The first method is agar diffusion, where the efficacy of each biopolymer against the tested microorganisms is measured by the diameter of the inhibition zone. The second method is microdilution which uses a microtiter plate (96-well plate) to determine the minimum inhibitory concentration (MIC) and bactericidal concentration (MBC). The inhibition zones and MICs of gentamicin and amphotericin B were also determined in parallel experiments to control the sensitivity of the test microorganisms. All tests were carried out in triplicate. We tested the antimicrobial activity of the tested extracts on:

✓ Two Gram-positive (G^+^) bacteria: *Staphylococcus aureus* ATCC 25923 (*S. Aureus*) and *Bacillus cereus* ATCC 14579 (*B. Cereus*);
✓ Two Gram-negative (G^−^) bacteria: *Escherichia coli* ATCC 35218 (*E. coli)* and *Salmonella Typhimurium* ATCC 14028 (*S. Typhim*).

These reference strains were obtained from the Laboratory for Analysis, Treatment and Recovery of Environmental Pollutants and Products (LATVPEP) at the Faculty of Pharmacy in Monastir, Tunisia.

To carry out the tests, we previously prepared five separate solutions of our biopolymers, at a concentration of 6 mg/mL, which will be noted as follows:

(B): CSDDA dissolved in 1% acetic acid;

(5): HTCC-5 dissolved in distilled water;

(6): HTCC-6 dissolved in distilled water;

(7): HTCC-7 dissolved in distilled water;

(8): HTCC-8 dissolved in distilled water.

(Ag/Cs NCs): Ag/Cs nanocomposite dissolved in distilled 1% acetic acid (AA) solution.

- *Agar diffusion method*

The antimicrobial activity of the biopolymer solutions was evaluated by the diffusion method on agar in disk. The microbial strains were revived and the turbidity adjusted to 0.5 McFarland, corresponding to 1–2 × 10^8^ CFU/mL for bacteria (OD = 0.08 to 0.1/λ = 625 nm). Filter paper disks (Wattman No. 1) of 6 mm diameter were individually impregnated with 10 μL of the solutions prepared at two different concentrations (cc_1_ = 1 mg/mL and cc_2_ = 6 mg/mL). These disks were then placed on the surface of agar media (Mueller–Hinton medium), previously inoculated with the test microorganisms. The Petri dishes were kept at 4 °C for 2 h before being incubated at 37 °C for 24 h. Afterwards, inhibition zone diameters (in mm) were measured. Gentamicin (15 μg/disk) served as positive control (T+).

ii. *Microdilution method: Determination of MIC and CMB*

The broth microdilution method was used to determine the minimum inhibitory concentration (MIC), according to the NCCLS-2012 recommendations [21]. Serial dilutions were prepared in a 96-well microtiter plate, covering a concentration range from 5 to 90% (*v*/*v*). The strains were adjusted to a final concentration of 5 × 10^5^ CFU/mL of bacteria and then added to each well. The bacteria were respectively incubated at 37 °C for 24 h.

The MIC is defined as the lowest concentration of the test material at which no visible growth of microorganisms is observed. Growth was determined by the appearance of turbidity in the broth. Gentamicin and amphotericin were used as positive controls. The minimum bactericidal concentration (MBC) corresponds to the lowest concentration of the biopolymer capable of eliminating 99.99% of bacteria after 18 to 24 h of contact with the antimicrobial. To determine the MBC, cultures were grown from the wells in which no growth was observed [22,23].

#### 2.2.10. Antiviral Activity

**Scheme 1:**
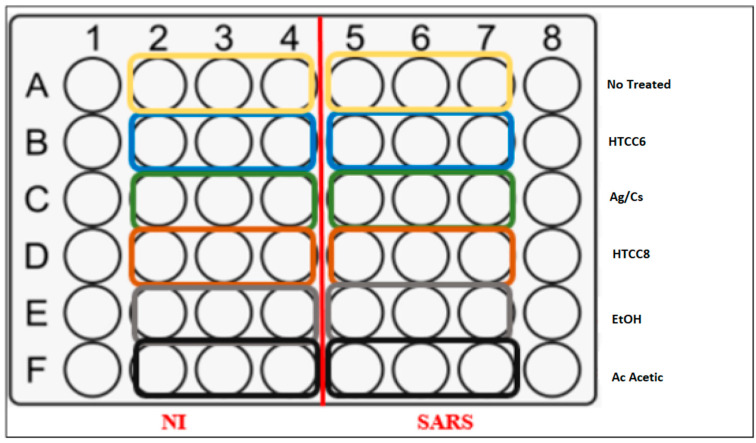
Well plates prepared for antiviral assays.

The evaluation of the antiviral activity of the selected HTCC biopolymers and Ag/Cs nanocomposite against SARS-Cov2 virus was carried out in the UB’L3 laboratory, TBMcore, University of Bordeaux, France. Three materials were tested—HTCC6, HTCC8 and Ag/Cs—according to the following protocol:

• In L2 (the day before), as described in Scheme 1 preparation of 48-well plates with 25,000 Vero cells in 200 μL of complete Dulbecco’s Modified Eagle Medium (DMEMc). Polymer solutions: 100 mg/mL, HTCC6 and HTCC8 56% EtOH/DMEMc and Ag/Cs 1% acetic acid/DMEMc.
• Deposit 18 μL of polymer solutions (final concentrations 6 mg/mL and 3.5% EtOH or 0.06% acetic acid after addition of the viral suspension) or 18 μL of equivalent solvents.
• At L3, 1 h after addition of the molecules, prepare a SARS viral suspension, with MOI = 0.01.
• Infect the cells with 100 μL of viral suspension or add 100 μL of DMEMc.
• Incubate for 2 h at 37 °C with 5% CO_2_.
• Remove all the liquid from the wells and add 200 μL of DMEMc per well.
• At L3 on day 3 post-infection:
  - Microscopic observation.
  - Measurement of cytopathic effect using a Celltox Green Cytotoxicity Assay (Promega).
  - Deposit 10 μL of lysis buffer into 2 wells of column 8 (positive control, 100% mortality).
  - Prepare the solution with 10 mL of assay buffer and 5 μL of CellTox™ Green Dye, adding 200 μL per well.
  - After 15 min in the dark, read the results on a Victor Nivo analyzer (HTDS, Massy, France) (485–500 nm Ex/520–530 nm Em).
  - Nucleic acid extraction with the Maxwell RSC 48 (Promega, Madison, WI, USA)
  - Add 300 μL of lysis buffer to 200 μL of supernatant and vortex for 10 s.
  - Add 30 μL of Proteinase K solution and vortex briefly. Incubate for 20 min at 56 °C.
  - In L1, RNA extraction was performed using a Maxwell RSC 48 according to the manufacturer’s recommendations. RT-qPCR was performed on the SARS-CoV-2 N gene and the B2M housekeeping gene.

#### 2.2.11. Cytotoxicity Assay

The cytotoxicity measurement technique we used is the MTT method [24], which is based on a colorimetric assay using the reagent of tetrazolium MTT (3-(4,5-dimethylthiazol-2-yl)-2,5-diphenyl tetrazolium bromide). This yellow compound has the particularity of being converted by a mitochondrial enzyme, succinate dehydrogenase, into a purple derivative: formazan. In the presence of MTT, the medium containing the living cells will be colored purple while the medium containing the dead cells will remain yellow. The MTT cytotoxicity test technique consists of depositing a series of ½ dilutions of an extract (100 μL) on confluent Vero cells in 96-well plates with a bottom. The starting concentration is 2.5 mg/mL which corresponds to a 3.75% ethanol concentration which is a non-toxic concentration for the cells. After 72 h, the dilutions were removed and then replaced with MTT (100 μL). After 3 h of incubation, the MTT solution is replaced with dimethyl sulfoxide (DMSO) to allow the dissolution of the formazan (15 min of contact). Then, the plate is introduced into a plate reader and the optical density (OD) of each well was read at a wavelength of 630 nm in comparison with that of the cell control. Thus, cytotoxicity is evaluated by determining its 50% cytotoxic concentration (CC50) which corresponds to the concentration giving 50% cell inhibition. The CC50 represents the concentration of the extract which gives an OD equal to half the OD of the cell control.

## 3. Results

### 3.1. Characterization of Deacetylated Chitosan

**Figure 1:**
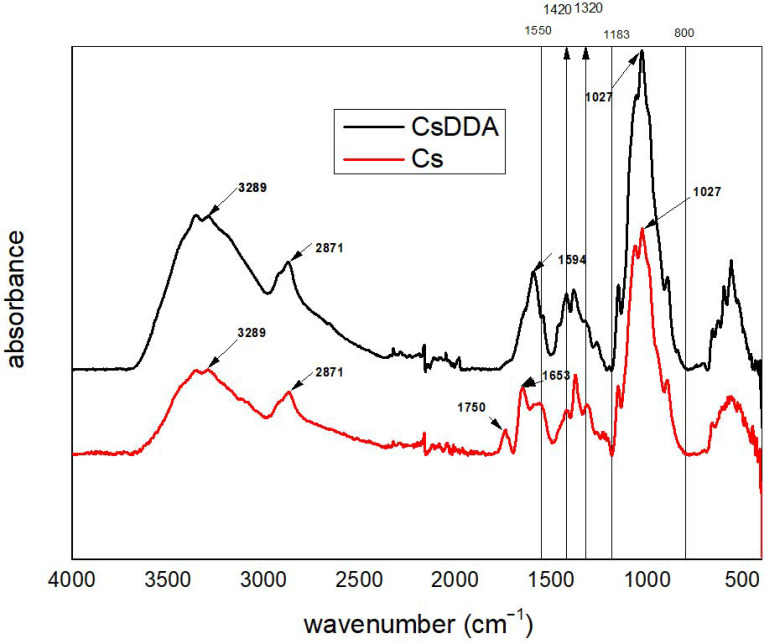
Comparison between FTIR spectra of Cs and CsDDA.

The FTIR spectra below (Figure 1) show a broad band at 3000–3700 cm^−1^ which is attributed to the stretching vibration of N-H and O-H bonds. The band at 2871 cm^−1^ corresponds to the stretching vibration of the C-H bond. The difference between the two spectra is the presence of the band at 1653 cm^−1^ and the band at 1550 cm^−1^ attributed respectively to amide I and amide II in the spectrum of Cs, which clearly shows that chitosan is not completely deacetylated. In addition, the band at 1594 cm^−1^, which corresponds to the NH_2_ bond, is relatively intense in the spectrum of CsDDA compared to the spectrum of Cs which confirms the increase in the degree of deacetylation of CsDDA compared to Cs. On the other hand, all the bands present between 1183 cm^−1^ and 800 cm^−1^ which correspond to the C-O-C and C-O bonds of the glycosidic cycle remain the same for the two polymers. These results are consistent with previous FTIR studies reported in the literature [25].

Adding the identification of new bonds or the disappearance of other bonds, the FTIR spectrum allows us to calculate the degree of deacetylation using Brugnerotto [26], Equation (2):

(2) $$\frac{A_{1320}}{A_{1420}}=0.3822+0.03133\times (100-DDA)\;\mathrm{with}\;R^{2}=0.99$$

Applying the values, the new DDA (90.67%) is much higher than the DDA (77.44%) before deacetylation in alkaline medium. The increase in DDA contributes significantly to the improvement of the biological activities of the polymer.

Also, proton NMR spectroscopy was used as an alternative characterization method to calculate the DDA of commercial chitosan Cs and chitosan after deacetylation CsDDA.

**Figure 2:**
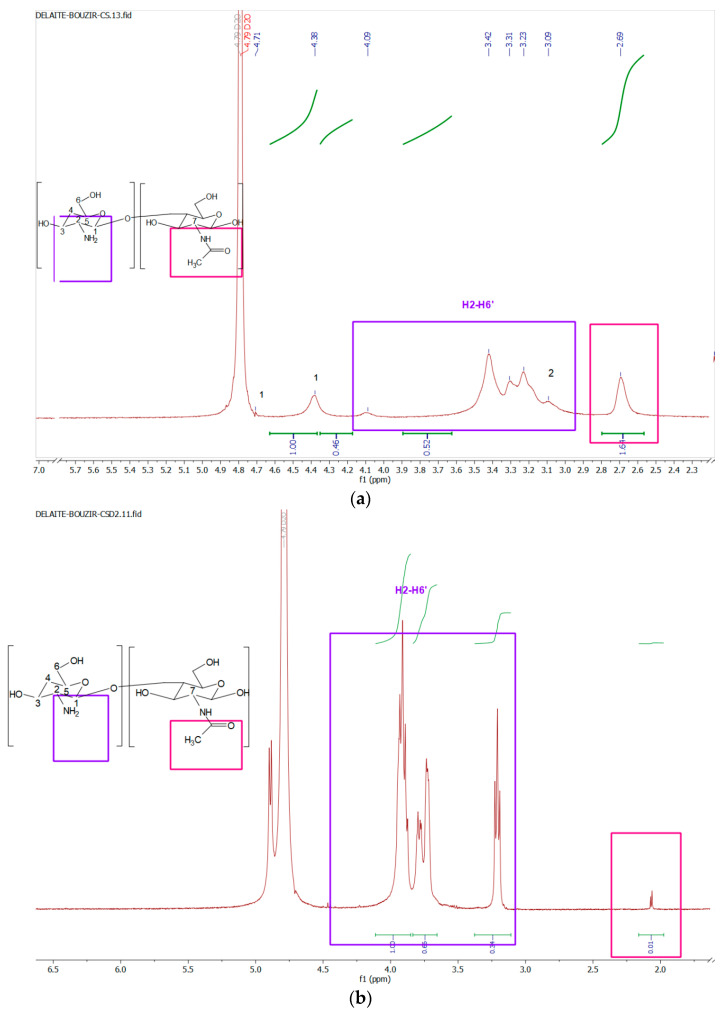
Proton NMR spectra of Cs (**a**) and CsDDA (**b**).

The spectrum of Figure 2a shows the different peaks of the as-received commercial chitosan. The anomeric H1 protons can be observed at high chemical shifts at 4.38 ppm and 4.71 ppm due to the orientation of the glycosidic bond relative to the ring oxygen [14]. The H2 proton of the amine is observed at 3.09 ppm. The H3, H4, H5, H6 and H6′ protons, which are linked to the six-ring, are observed between 3.2 and 4.1 ppm, producing a broad signal in the middle of the spectrum due to their similar electron densities. The H7 proton of the N-acetylglucosamine unit is observed at 2.69 ppm. Then, the spectrum in (Figure 2b) above describes the deacetylation reaction carried out for commercial chitosan. It is clearly noted that the H7 proton of the N-acetylglucosamine unit, observed in the spectrum in Figure 2a, has decreased and tended towards 0, whereas the H2 proton of the amine located at 3.21 ppm has become more intense. Finally, the H3, H4, H5, H6 and H6′ protons, which are linked to the six-ring, are still located in the same location between 3.2 ppm and 4.1 ppm.

The deacetylation degree was determined by applying the following Equation (3) [27,28]:

(3) $$DDA(\%)=[1-\frac{\frac{I_{CH3}}{3}}{\frac{I(H2-{H6}^{\prime })}{6}}]\times 100$$

Applying this Equation (3) for Cs and CsDDA we obtain DDA values of 74.68% and 94% respectively. This confirms the deacetylation reaction carried out. These results are close to those calculated from the FTIR spectrum. The degree of deacetylation of chitosan is very important since it conditions both the number of free amines that can react with target molecules and its solubility in an aqueous medium: the more it is deacetylated, the more soluble it will be.

### 3.2. Characterization of HTCC Polymers and Ag/Cs Nanocomposite

#### 3.2.1. SEM of Modified Chitosan Materials

**Figure 3:**
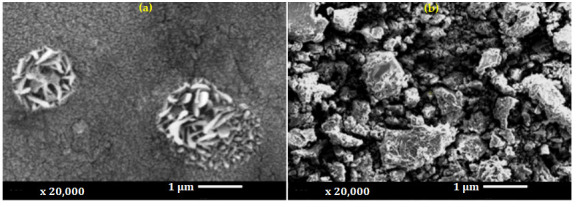
Morphological analysis of (**a**) AG/Cs nanocomposite and (**b**) HTCC6 polymer.

The SEM image below acquired at a magnification of ×20,000 (Figure 3a) reveals the morphology of the chitosan/silver nanoparticles. While some earlier studies described Ag nanoparticles as smooth and essentially non-porous chitosan spheres, other reports have highlighted a markedly porous surface morphology [18]. Our findings are consistent with the latter, as the spheres produced in this study also display a porous structure. In addition, the chitosan matrix exhibits a granular and slightly cracked surface texture, which is typically associated with the drying process. Bright structures observed on the surface correspond to silver nanoparticles, mostly within the nanometric size range (40–110 nm), although larger agglomerates can also be seen. Some particles display elongated or irregular shapes; this result is in agreement with the observations described in reference [29] and it indicates that the majority of Ag nanoparticles within the composite are indeed spherical.

The SEM micrograph presented in Figure 3b illustrates the surface morphology of quaternized chitosan derivatives (HTCCs), revealing structural characteristics typically associated with chitosan polymers modified by glycidyltrimethylammonium chloride (GTMAC) grafting. In comparison with unmodified chitosan, the HTCC samples exhibit a noticeably denser and more compact surface morphology.

This compact appearance suggests an even distribution of quaternary ammonium groups along the chitosan macromolecular backbone. Such morphological evolution can be attributed to the chemical modification of the amino groups, which reduces inter- and intramolecular hydrogen bonding while promoting electrostatic repulsion between positively charged polymer chains [30]. These combined effects enhance chain mobility, limit aggregation, and lead to a more regular and stabilized polymeric arrangement [31].

From an application perspective, this morphology is particularly advantageous for coating processes, as it favors uniform surface coverage and stable interfacial contact with textile substrates. Overall, the SEM observations in Figure 3b provide convincing morphological evidence of successful chitosan quaternization and are fully consistent with the expected physicochemical properties, increased charge density, and improved biological performance reported for HTCC materials.

#### 3.2.2. FTIR Analysis

**Figure 4:**
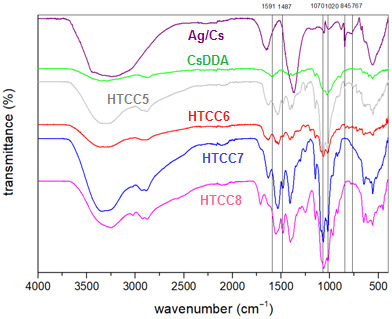
FTIR spectra of CsDDA, HTCC (5, 6, 7, 8) and Ag/Cs.

Figure 4 shows the spectra of unmodified deacetylated chitosan CsDDA and HTCC (5, 6, 7, 8) with different degrees of substitution following the addition of different volumes of GTMAC (5, 6, 7, 8) mL during chitosan modification processes, as well as the Ag/Cs nanocomposite.

Regarding the Ag/Cs nanocomposite, the silver nanoparticles do not exhibit characteristic bands in infrared spectroscopy, but interactions with the functional groups can lead to the appearance of new bonds, such as the band around 845 cm^−1^ [29].

This spectrum shows the most significant difference between the spectrum of unmodified chitosan and that of HTCC. This major difference is the appearance of a new band at 1487 cm^−1^, which corresponds to the angular deformation of the C-H group, and this verifies the presence of the quaternary ammonium salt [32]. Furthermore, the primary amine N-H bond present in unquaternized chitosan at 1590 cm^−1^ disappeared in the spectrum of quaternized chitosan HTCC (5, 6, 7, 8) [16,29,32].

The characteristic bands of chitosan, which correspond to the polysaccharide and pyranose structure, remained present even after modification. The characteristic bands of primary and secondary alcohol did not change in the spectra of modified and unmodified chitosan at 1082 cm^−1^. This proves that the modification of chitosan is well established at the NH_2_ sites [33].

#### 3.2.3. ^1^H NMR Spectroscopy of Modified Chitosan Materials

**Figure 5:**
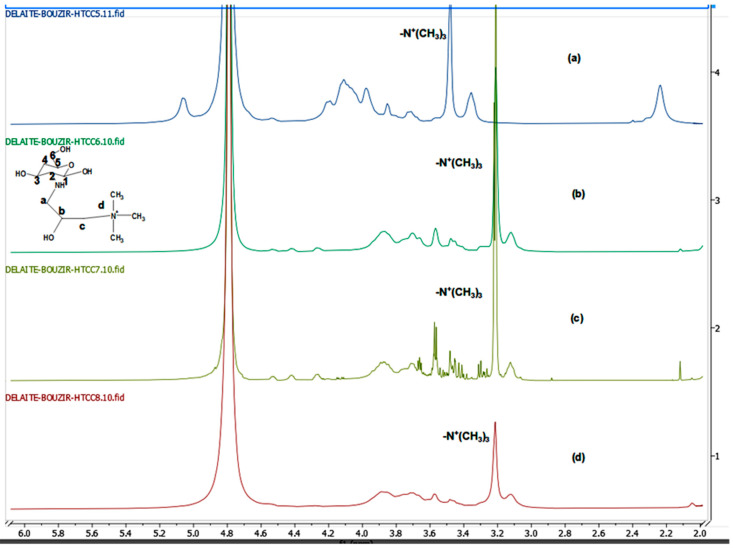
^1^H NMR spectra of (**a**) HTCC5, (**b**) HTCC6, (**c**) HTCC7, (**d**) HTCC8.

The analysis of quaternized chitosan by proton NMR allowed the following spectra to be plotted (Figure 5a–d) to identify the new peaks which appeared after the chitosan modification reaction and thus to confirm the synthesis of HTCC (5, 6, 7, 8) respectively [34].

Comparing the proton NMR spectrum of chitosan with that of HTCCs (5, 6, 7, 8), we note the appearance of an intense peak around 3.2 ppm which corresponds to the N^+^(CH_3_)_3_ group [35] while preserving the characteristic skeleton of the starting polymer (H1, H2, H3, H4, H5, H6).

The signals observed between 3.3 and 3.6 ppm in Figure 5c are attributed to minor residual low-molecular-weight species, likely originating from traces of ethanol used during purification and/or small amounts of unreacted GTMAC. These minor signals do not interfere with the assignment of the main polymer peaks, particularly the characteristic signal at ~3.2 ppm corresponding to the quaternary ammonium groups.

Table 1 summarizes all the characteristic peaks of each HTCC, indicating the position of each proton.

These results obtained are in good agreement with those mentioned in the literature [15,36].

#### 3.2.4. Conductometric Titration

**Figure 6:**
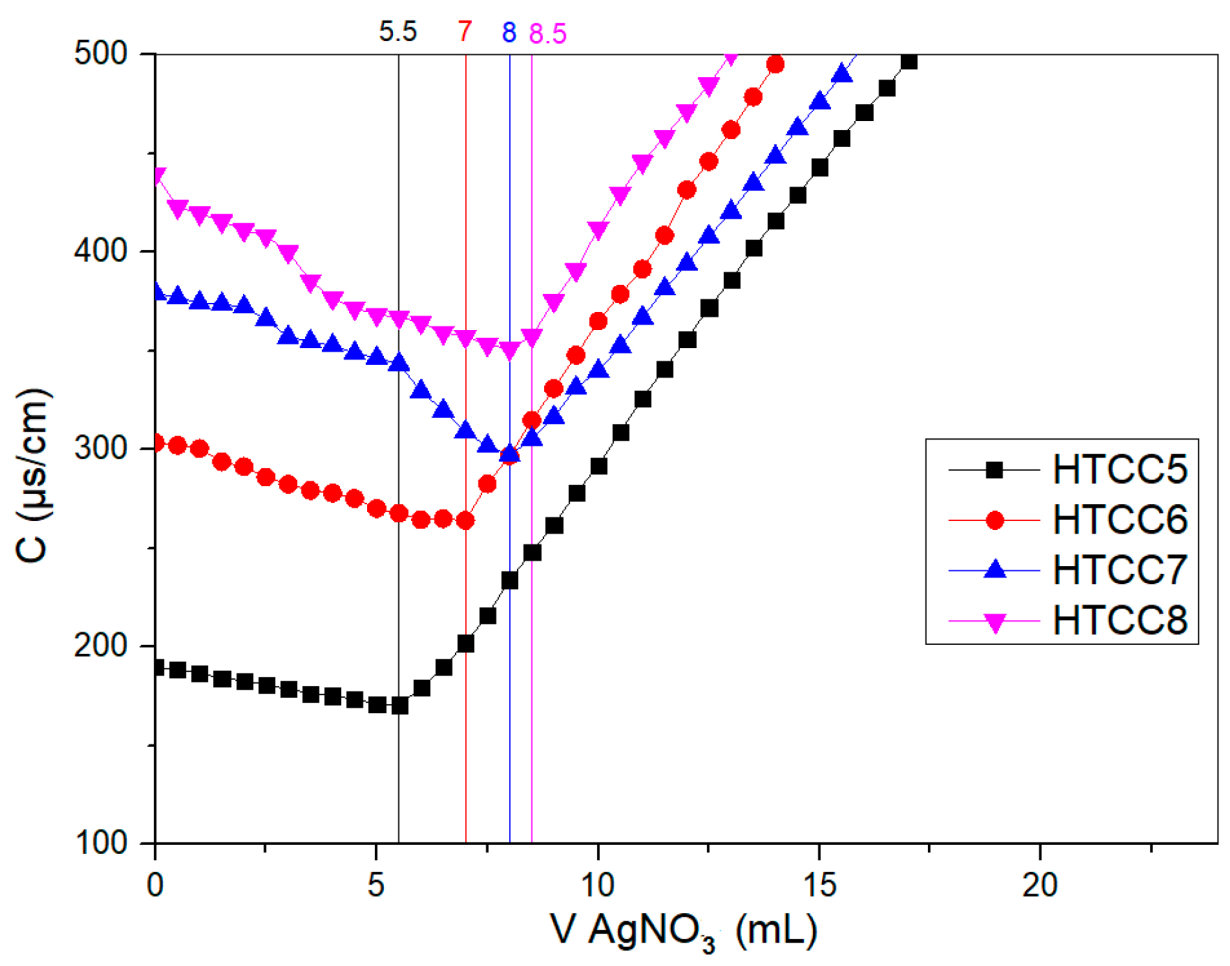
Conductometric titration of HTCC (5, 6, 7, 8).

From Figure 6, we can extract the volumes of AgNO_3_ at equivalence for each HTCC and then we can calculate QD by applying the formula in Equation (1) [37].

Table 2 below describes the obtained results.

It is noted that as the volume of GTMAC used during chitosan modification increases, the volume of AgNO_3_ increases, hence the degree of quaternization (QD) increases. This confirms the chitosan modification reaction and the substitution of amino groups with quaternary groups. Although AgNO_3_ consumption increases with the amount of GTMAC, it is not strictly proportional to the degree of quaternization (QD), because the reaction is limited by the number of reactive amino sites on the HTCC polymer.

#### 3.2.5. Thermal Stability

**Figure 7:**
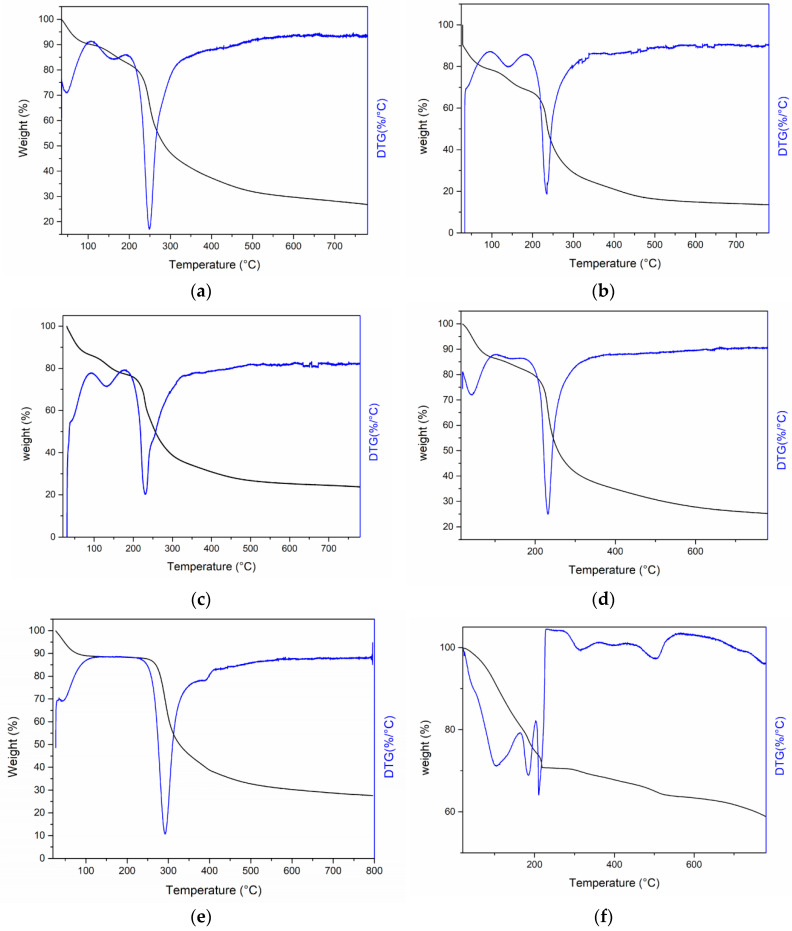
TGA and DTG curves recorded over the temperature range of 25 °C to 700 °C spectrum of (**a**) HTCC5, (**b**) HTCC6, (**c**) HTCC7, (**d**) HTCC8, (**e**) CsDDA and (**f**) Ag/Cs.

Chitosan CsDDA exhibits two remarkable phases of mass loss during heating. The first phase is located between approximately 40 °C and 100 °C during which desorption has caused a mass loss of 10% of its initial mass. The second phase is located between approximately 270 °C and 320 °C. This phase is related to the degradation of the biopolymer and the vaporization of volatile compounds. The maximum rate is 290 °C as shown in the DTG curve (Figure 7e). Another degradation step, which is generally attributed to the carbonation of residual carbon and the pyranose cycle, is located between 300 °C and 500 °C, while the maximum rate is around 387 °C as seen in the DTG curve (Figure 7e). Then, CsDDA continues the mass loss process up to 72% of its initial mass at 700 °C.

For the HTCCs (5, 6, 7, 8), they exhibited almost the same thermal behavior despite the difference in the QDs. However, in the first phase, they lost almost 15% of their initial mass between 50 °C and 100 °C. Furthermore, the temperature of the second phase was reduced to approximately 230 °C, which proves that the modification reduced the thermal stability of the biopolymer since the functional groups of the quaternary ammonium salt weaken the molecular interaction (hydrogen bonds) along the polymer chain. Similarly, the HTCCs lost 75% to 85% of their initial mass at 700 °C [35].

Regarding the Ag/Cs nanocomposite, we note that the effect of silver nanoparticles is visible in the reduction in the mass loss of the composite to 41% of its initial mass at 800 °C. Therefore, silver nanoparticles play a very important role in thermal stability and this makes sense since it is a metal [29]. Moreover, the degradation phases of chitosan are still visible in the ATG spectrum of the nanocomposite.

### 3.3. Antimicrobial Activities of HTCC and Ag/CS Nanocomposite

- *Agar diffusion method*

Table 3 shows the diameters of the inhibition zones (in mm) observed after 24 h of incubation at 37 °C to evaluate the antibacterial activity of crude chitosan (B) and different modified forms of chitosan (HTCC5, HTCC6, HTCC7, HTCC8, and Ag/Cs) by the agar diffusion method for C1 = 1 mg/mL.

Crude chitosan (B) demonstrated measurable antibacterial activity against several bacterial strains, with inhibition zones of 13 mm for *Escherichia coli*, 16.33 mm for *Pseudomonas aeruginosa*, 14.66 mm for *Salmonella Typhimurium*, and 12.55 mm for *Bacillus cereus*. However, no inhibitory effect was observed against *Staphylococcus aureus*, suggesting a limited interaction with this Gram-positive strain. After correction for solvent effects, a decrease in inhibition zone diameters was noted, indicating that the actual antibacterial activity of crude chitosan is lower than the raw values suggest. In contrast, none of the modified chitosan forms (HTCC5, HTCC6, HTCC7, HTCC8) exhibited antibacterial activity against any of the tested bacteria. This loss of activity may be attributed to structural modifications that impair the polymer’s ability to interact with bacterial membranes. Notably, the Ag/Cs nanocomposite exhibited pronounced antibacterial activity against all tested strains, with inhibition zones ranging from 14.66 mm (*E. coli*) to 19.66 mm (*B. cereus*), outperforming the other chitosan-based polymers. All experiments were performed in triplicate (n = 3). This enhanced effect, compared to crude chitosan, indicates that the incorporation of silver nanoparticles substantially improves antibacterial performance. Furthermore, *S. aureus*, which was resistant to crude chitosan, was inhibited by Ag/Cs (15.33 mm), confirming the broad-spectrum efficacy of silver nanoparticles, including against Gram-positive bacteria.

Table 4 presents the diameters of the inhibition zones (in mm) observed after 24 h of incubation at 37 °C to evaluate the antibacterial activity of crude chitosan (B) and different modified forms of chitosan (HTCC5, HTCC6, HTCC7, HTCC8, and Ag/Cs) by the agar diffusion method for a (C2 = 6 mg/mL) concentration of the polymers.

Increasing the chitosan concentration from 1 mg/mL (C1) to 6 mg/mL (C2) resulted in a significant enhancement of its antibacterial activity. While C1 showed limited or no inhibitory effect against several tested strains, the higher concentration at C2 led to markedly larger inhibition zones, particularly against *Pseudomonas aeruginosa* (19 mm) and *Escherichia coli* (15.33 mm). This concentration-dependent trend was also observed with chitosan derivatives, especially HTCC6 and HTCC7, which exhibited no activity at cc_1_ but demonstrated notable antibacterial effects at C2. At this concentration, HTCC6 was particularly effective against *Bacillus cereus* (18 mm) and *Staphylococcus aureus* (16.66 mm). Moreover, the formulation combining chitosan with silver nanoparticles (Ag/Cs) showed the strongest antibacterial activity overall, with inhibition zones reaching up to 23.66 mm against *Bacillus cereus*, indicating a potentiated synergistic effect. These findings clearly support a dose-dependent antibacterial efficacy of chitosan and its derivatives, likely due to improved interaction with bacterial cell membranes at higher concentrations.

**Figure 8:**
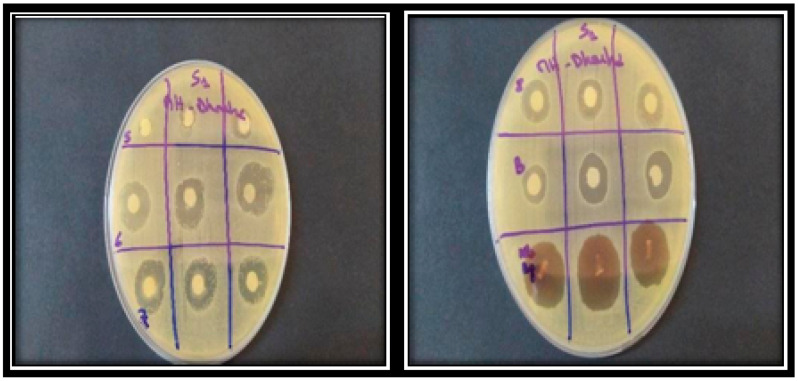
Antibacterial activity of crude chitosan B, modified chitosans (5, 6, 7 and 8), and Ag/Cs for (C2 = 6 mg/mL) against *Staphylococcus aureus* ATCC 25923.

Figure 8 below illustrates a representative example of antibacterial activity assessed using the agar diffusion method; inhibition zones were measured based on the diameters surrounding the applied disks.

ii. *Microdilution method: Determination of MIC and CMB*

Table 5 below describes the results obtained after performing the microdilution. A marked reduction in MIC and MBC values was observed for all water-soluble modified chitosan, except for HTCC5 with a low degree of substitution (DS = 42%), which led to increased MIC and MBC values against both Gram-positive and Gram-negative strains. In contrast, HTCC6, HTCC7, and HTCC8 showed minimal variation in antimicrobial activity, likely due to their similar DS values. Furthermore, the nanocomposite exhibited the lowest MIC and MBC values among all tested formulations. The nanocomposite exhibits enhanced activity, which may be attributed to the sizes of the silver nanoparticles, providing a substantially higher surface area compared with the bulk polymer.

### 3.4. Antiviral Activity and Cytotoxicity

All three compounds maintained cell viability above 70% up a concentration of 6 mg/mL, indicating no significant cytotoxic effects. Consequently, this concentration was used for the antiviral activity tests.

**Figure 9:**
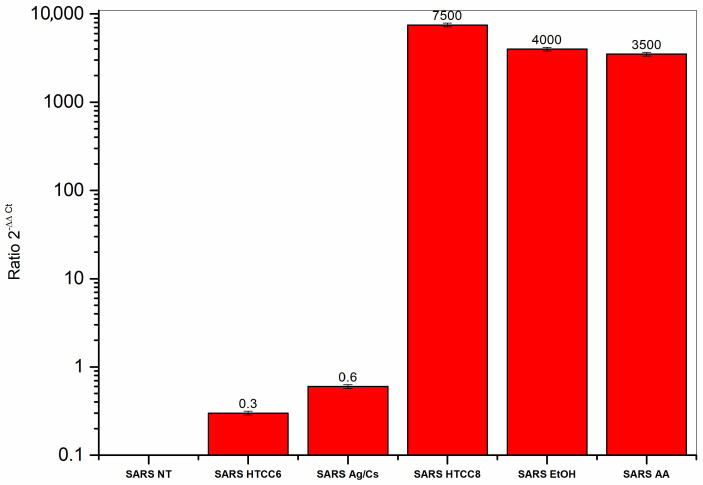
SARS-CoV-2 gene expression (2^−ΔΔCt^) in response to different polymers and solvents.

Figure 9 depicts the relative expression levels of SARS-CoV-2 genes following exposure to different polymers, as calculated using the 2^−ΔΔCt^ method. Ct values for the viral N gene and the reference GAPDH gene were determined under each experimental condition, and the resulting ratio reflects the viral infection level normalized to an equivalent cell number.

A value equal to one corresponds to a viral RNA amount equivalent to that of the infected, untreated control cells. Values greater than one indicate an increased level of viral RNA, whereas ratios lower than one reflect a decrease in the detected viral RNA, suggesting a potential antiviral effect of the tested polymers.

Compounds C1 (HTCC6) and C2 (Ag/Cs) exhibited significant antiviral activity, with C1(HTCC6) inducing a moderate reduction in viral RNA and C2 (Ag/Cs) causing a more pronounced decrease, whereas C4 (HTCC8) showed no effect, highlighting the role of polymer substitution degree in determining antiviral efficacy.

**Figure 10:**
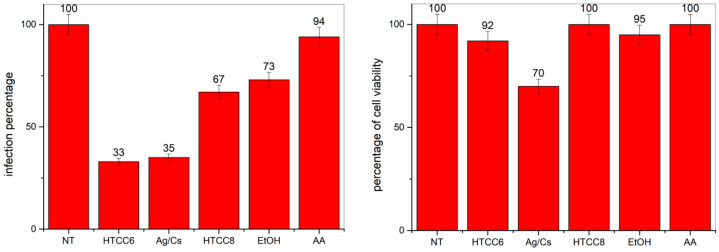
Cell viability and infection levels in Vero cells treated with the tested compounds HTCC6, Ag/Cs and HTCC8. The right panel shows the percentage of viable cells, and the left panel displays the percentage of infected cells.

Moreover, the CellTox assay showed (Figure 10) a markedly lower infection rate in Vero cells treated with compounds C2 (33%) and C1 (35%), whereas compound C4 produced less convincing results (67%). Cell viability measurements were consistent across all compounds and aligned with the cytotoxicity data obtained with CellTiter, as well as with the RT-qPCR findings regarding antiviral activity.

## 4. Discussion

The findings of this study further confirm the strong antimicrobial potential of chitosan and its quaternized derivatives. In particular, HTCC exhibited pronounced antiviral activity against enveloped Coronaviruses, including SARS-CoV-2, through interactions with the viral spike protein that hinder viral attachment to host cell receptors, as previously reported in the literature [38]. This targeted antiviral mechanism highlights the relevance of HTCC as a functional polymer capable of inhibiting viral entry without the need for nanoparticle-based systems, which may offer advantages in terms of formulation simplicity and biocompatibility.

In parallel, chitosan-based composites incorporating silver nanoparticles have been widely reported to display enhanced antibacterial and antiviral performance against respiratory viruses as well as Gram-positive and Gram-negative bacteria. In these systems, silver nanoparticles are known to generate reactive oxygen species and disrupt microbial membranes, while the chitosan matrix acts as a stabilizing and biocompatible carrier that promotes interaction with biological surfaces, thereby improving overall antimicrobial efficacy [39]. Together, these polymeric and composite approaches illustrate complementary strategies for the design of broad-spectrum antimicrobial materials.

Beyond material composition, the antimicrobial performance of HTCC is strongly influenced by its degree of substitution (DS). Notably, increasing the amount of GTMAC led to a higher degree of substitution of HTCC, highlighting the direct effect of reagent volume on modification efficiency. Our antibacterial results for *S. aureus* and other bacterial strains were therefore compared with previously reported data [35], where increasing the DS from 59% to 79% led to a significant reduction in the minimum inhibitory concentration (MIC), whereas a further increase to 93% did not provide additional benefits for *E. coli* or *S. aureus*. Consistent with these observations, our study revealed a marked improvement in MIC values for *S. aureus* when the DS increased from 42% to 74%, followed by a plateau between 74% and 83%, indicating a non-linear relationship between DS and antibacterial activity. A similar trend was observed for *P. aeruginosa*, *Salmonella Typhimurium*, and *B. cereus,* for which no further improvement in MIC was detected beyond a DS of 74%. In contrast, *E. coli* exhibited a distinct response, with MIC values remaining unchanged between DS values of 42% and 74.39%, followed by a gradual improvement when the DS increased from 74.76% to 83%. This behavior suggests that certain Gram-negative bacteria may require a higher threshold of substitution to achieve optimal inhibition.

A comparable dependence on DS was also evident for antiviral activity. In contrast to the study by Kerwald et al. [38], which reported antiviral effects for all tested HTCCs across a DS range of 59–93%, more mechanistic investigations have demonstrated that antiviral efficacy against SARS-CoV-2 is closely linked to the polymer structure, with HTCCs exhibiting intermediate DS values (approximately 57–77%) showing particularly strong inhibition in vitro and ex vivo [40]. In agreement with these findings, our results indicate that antiviral activity is maximized at a DS of 74%, whereas no detectable antiviral effect was observed for a higher DS of 83%. This suggests the existence of an optimal substitution window, beyond which excessive cationic charge density or conformational constraints of the polymer chains may hinder effective interactions with viral surface proteins.

Overall, the combined analysis of HTCC, chitosan–silver composites, and DS-dependent effects underscores the versatility of chitosan-based materials as tunable platforms for antimicrobial applications. Fine control of the degree of substitution emerges as a critical parameter for balancing targeted antiviral activity, broad-spectrum antibacterial performance, and overall material efficiency, depending on the intended biomedical application.

## 5. Conclusions

In this study, a series of chemically modified chitosan-based materials were developed for enhanced antibacterial and antiviral performance in advanced medical textile applications. Quaternization with glycidyltrimethylammonium chloride and incorporation of silver nanoparticles enabled the design of functional polymers and nanocomposites with distinct and complementary bioactive properties.

Biological assessments showed that the Ag/Cs nanocomposite exhibited strong broad-spectrum antibacterial activity and enhanced thermal stability, while HTCC6 displayed significant antiviral activity against SARS-CoV-2 with low cytotoxicity. These results highlight the complementary bioactive properties of the modified chitosan derivatives and their potential for functionalization of medical textiles.

In addition, establishing links between the applied chemical modification approaches and the observed biological responses offers deeper understanding for the rational design of chitosan-based systems. In particular, factors such as cationic charge density and the organization of nanocomposite structures appear to play a decisive role in both microbial and viral inhibition. These insights further support the relevance of tailored chitosan derivatives as efficient and sustainable candidates for next-generation biomedical textile applications.

Although the nanocomposite displayed strong antimicrobial efficacy, quaternized chitosan offers additional advantages, including improved aqueous solubility and reduced cytotoxicity. These characteristics make HTCC derivatives especially attractive for potential future applications in medical textile functionalization, where processability, safety, and durability are important considerations. From a sustainability perspective, the use of bio-based chitosan derivatives represents a promising alternative to conventional antimicrobial agents for infection prevention materials.

Overall, the findings of this work provide valuable insights into the structure–activity relationships governing the antibacterial and antiviral behavior of modified chitosan systems. The developed materials constitute a robust platform for the next generation of functional medical textiles. Future investigations will focus on their application as durable textile coatings, evaluation under realistic use conditions, and the optimization of nanostructured architectures to further enhance antiviral efficiency and long-term performance.

## Figures and Tables

**Table 1:** Peak assignment of ^1^H NMR spectra of HTCC samples.

*Proton Position*	*HTCC5*	*HTCC6*	*HTCC7*	*HTCC8*
***H_a_***	*3.35*	*3.12*	*3.12*	*3.12*
***H_b_***	*4.54*	*4.42*	*4.54*	*4.26*
***H_c_***	*3.35*	*3.30*	*3.30*	*3.48*
***H_d_***	*3.49*	*3.21*	*3.21*	*3.21*
***H_1_***	*5.07*	*4.53*	*4.71*	*4.54*
***H_2_***	*2.24*	*2.12*	*2.12*	*2.05*
***H_3_***	*3.85*	*3.87*	*3.89*	*3.87*
***H_4_***	*3.98*	*3.71*	*3.72*	*3.75*
***H_5_***	*4.19*	*3.65*	*3.66*	*3.71*
***H_6_***	*4.11*	*3.57*	*3.58*	*3.58*

**Table 2:** QD values obtained from conductometric titration method.

HTCC	HTCC5	HTCC6	HTCC7	HTCC8
V AgNO_3_ (mL)	5.5	7	8	8.5
QD (%)	42.2	74.5	74.7	83

**Table 3:** Diameters of inhibition zones showing the antibacterial activity of crude chitosan (B) and modified chitosan (HTCC5, HTCC6, HTCC7, HTCC8, Ag/Cs) by the agar diffusion method (for C1 = 1 mg/mL).

Diameter of the Inhibition Zone (in mm) After 24 h of Incubation at 37 °C									
Microorganism	Origin	B	HTCC5	HTCC6	HTCC7	HTCC8	Ag/Cs	AA 1%	T+
***Staphylococcus aureus***	ATCC 25923	-	-	-	-	-	15.33 ± 0.47	-	30
***Escherichia coli***	ATCC 35218	13	-	-	-	-	14.66 ± 0.47	-	30
***Pseudomonas aeruginosa***	ATCC 27853	16.33 ± 0.94	-	-	-	-	17.33 ± 0.94	10.66 ± 0.47	30
Corrected value		5.67 ± 0.47	-	-	-	-	6.67 ± 0.47	-	30
***Salmonella Typhimurium***	ATCC 14028	14.66 ± 0.47	-	-	-	-	18.66 ± 0.47	12.66 ± 0.47	25
Corrected value		2	-	-	-	-	6	-	25
***Bacillus cereus***	ATCC 14579	12.55 ± 1.55					19.66 ± 0.62	12.33 ± 0.47	30
Corrected value		0.22 ± 0.08	-	-	-	-	7.33 ± 0.15	-	30

**Table 4:** Diameters of inhibition zones showing the antibacterial activity of crude chitosan (B) and modified chitosan (HTCC5, HTCC 6, HTCC 7, HTCC 8, Ag/Cs) by the agar diffusion method (for C2 = 6 mg/mL).

Diameter of the Inhibition Zone (in mm) After 24 h of Incubation at 37 °C									
Microorganism	Origin	B	HTCC5	HTCC6	HTCC7	HTCC8	Ag/Cs	AA1%	T+
***Staphylococcus aureus***	ATCC 25923	11.66 ± 0.47	10	16.66 ± 2.49	14.33 ± 0.47	12	17.33 ± 0.47	-	30
***Escherichia coli***	ATCC 35218	15.33 ± 5.43	-	10	10	10.33 ± 0.47	16.66 ± 0.94		23
***Pseudomonas aeruginosa***	ATCC 27853	19 ± 3.26	-	12	12.33 ± 1.24	-	22.66 ± 0.47	10.66 ± 0.47	23
Corrected value		8.34 ± 2.79	-	12	12.33 ± 1.24	-	12	-	30
***Salmonella Typhimurium***	ATCC 14028	14.66 ± 0.47	-	12	-	12	20.66 ± 0.94	12.66 ± 0.47	25
Corrected value		2	-	-	-	-	8 ± 0.47	-	25
***Bacillus cereus***	ATCC 14579	12.66 ± 0.47	11 ± 0.81	18 ± 1.41	17.66 ± 0.47	13	23.66 ± 2.62	12.33 ± 0.47	29
Corrected value		0.33	11 ± 0.81	18 ± 1.41	17.66 ± 0.47	13	11.33 ± 2.15		29

**Table 5:** Minimum inhibitory concentration (MIC) and bactericidal concentration (MBC) of modified chitosan (HTCC5, HTCC6, HTCC7, HTCC8, Ag/Cs) and crude chitosan (B) expressed in (mg/mL).

Microorganism	Samples	MIC	MBC
***Staphylococcus aureus*** **ATCC25923**	HTCC5	3.9	4.5
	HTCC6	0.3	0.3
	HTCC7	0.3	1.8
	HTCC8	0.3	1.2
	Ag/Cs	0.3	0.3
	Ag/Cs(corrected value)	0.6	0.6
	B	0.3	0.3
	B(corrected value)	0.6	0.6
	AA 1%	0.3	0.3
***Escherichia coli*** **ATCC35218**	HTCC5	3.9	3.9
	HTCC6	3.9	3.9
	HTCC7	1.5	3
	HTCC8	0.3	0.6
	Ag/Cs	0.3	0.3
	Ag/Cs(corrected value)	0.6	0.6
	B	1.5	1.5
	B(corrected value)	1.8	1.8
	AA 1%	0.3	0.3
***Pseudomonas aeruginosa*** **ATCC27853**	HTCC5	3.3	4.8
	HTCC6	0.3	4.5
	HTCC7	0.6	3.3
	HTCC8	0.3	3
	Ag/Cs	0.3	0.3
	Ag/Cs(corrected value)	0.6	0.6
	B	0.3	0.6
	B(corrected value)	0.6	0.9
	AA 1%	0.3	0.3
***Salmonella Typhimurium*** **ATCC14028**	HTCC5	4.8	5.1
	HTCC6	0.3	0.3
	HTCC7	0.3	1.8
	HTCC8	0.3	1.8
	Ag/Cs	0.3	0.3
	Ag/Cs(corrected value)	0.6	0.6
	B	0.3	0.3
	B(corrected value)	0.6	0.6
	AA 1%	0.3	0.3
***Bacillus cereus*** **ATCC14579**	HTCC5	5.1	5.7
	HTCC6	0.3	5.7
	HTCC7	0.3	5.7
	HTCC8	0.3	5.7
	Ag/Cs	0.3	0.3
	Ag/Cs(corrected value)	0.6	6
	B	5.1	5.7

## Data Availability

The original contributions presented in this study are included in the article. Further inquiries can be directed to the corresponding authors.
